# Granular Hall Sensors for Scanning Probe Microscopy

**DOI:** 10.3390/nano11020348

**Published:** 2021-02-01

**Authors:** Roland Sachser, Johanna Hütner, Christian H. Schwalb, Michael Huth

**Affiliations:** 1 Institute of Physics, Goethe University, Max-von-Laue-Str. 1, 60438 Frankfurt am Main, Germany; sachser@physik.uni-frankfurt.de; 2 GETec Microscopy GmbH, Am Heumarkt 13, 1030 Wien, Austria; johanna.huetner@getec-afm.com; 3 Quantum Design Microscopy, Im Tiefen See. 60a, 64293 Darmstadt, Germany; schwalb@qd-microscopy.com

**Keywords:** focused electron beam induced deposition, granular ferromagnets, scanning Hall probe microscopy

## Abstract

Scanning Hall probe microscopy is attractive for minimally invasive characterization of magnetic thin films and nanostructures by measurement of the emanating magnetic stray field. Established sensor probes operating at room temperature employ highly miniaturized spin-valve elements or semimetals, such as Bi. As the sensor layer structures are fabricated by patterning of planar thin films, their adaption to custom-made sensor probe geometries is highly challenging or impossible. Here we show how nanogranular ferromagnetic Hall devices fabricated by the direct-write method of focused electron beam induced deposition (FEBID) can be tailor-made for any given probe geometry. Furthermore, we demonstrate how the magnetic stray field sensitivity can be optimized in situ directly after direct-write nanofabrication of the sensor element. First proof-of-principle results on the use of this novel scanning Hall sensor are shown.

## 1. Introduction

Scanning-probe based imaging of magnetic stray field distributions above a ferromagnetic sample can be accomplished by several complementary techniques, such as magnetic force microscopy (MFM) [1], scanning SQUID microscopy (SSQM) [2,3] and scanning Hall probe microscopy (SHPM) [4,5]. Each of these techniques has particular strengths and weaknesses. The most relevant figure of merit for each of these scanning approaches is the smallest magnetic stray field change detectable in conjunction with the achievable lateral resolution. Generally speaking, SSQM excels in stray field sensitivity but is limited regarding the lateral resolution. MFM can reach lateral resolutions in the 10 nm range with good stray field sensitivity. However, the extraction of absolute stray field magnitude is challenging. SHPM with a two-dimensional electron gas (2DEG) [6] or semi-metal (typically Bi) [4,7] as sensor material provides a good combination of stray field sensitivity and lateral resolution with the added advantage of being a non-invasive probe type, i.e., the probe does not produce a stray field of its own, which may cause changes of the magnetization distribution in the sample. SHPM with a granular ferromagnet (GFM) as sensor material has the potential to exhibit the same advantages as the semi-metal probes but at a significantly improved stray field sensitivity, albeit at the cost of introducing a very small—typically negligible due to shape anisotropy—stray field of its own. This is due to the anomalous or extraordinary Hall effect (EHE) observed in ferromagnetic materials, which is additive to the conventional or ordinary Hall effect (OHE) of metals. Expressed via the sample geometry independent Hall resistivity $\rho_{H}$ one finds [8]

(1) $$\rho_{H}=\rho_{OHE}+\rho_{EHE}=\mu_{0}\left(R_{0}H+R_{S}M\left(T,H\right)\right)$$

with *H* representing the external magnetic field, M(T,H) the magnetic field and temperature-dependent magnetization and $R_{0}$ and $R_{S}$ as material-dependent constants fulfilling the relation $R_{S}\gg R_{0}$. The strongly enhanced Hall effect in ferromagnets is due to both an intrinsic contribution related to the electronic band structure and extrinsic contributions stemming from chiral scattering contributions caused by non-magnetic impurities; see [8] for a recent review. Importantly, for granular ferromagnets, i.e., magnetic nanoparticles embedded into a dielectric matrix, a very pronounced increase in the EHE can result, depending on the material- and microstructure-dependent volume fraction of the ferromagnetic metallic component [9]. In addition, due to the granular structure, a cooperative ferromagnetic state of the material is only observed below a characteristic blocking temperature $T_{B}$ for which $T_{B}\ll 300$ K is essential regarding sensor applications. Namely, for $T>T_{B}$ super-paramagnetic behavior of the GFM does guarantee a hysteresis-free dependence of the EHE on the applied stray field and a linear field dependence as long as the field strength remains sufficiently below the saturation field.

With a view of scanning Hall probe applications, the strongly enhanced EHE is very beneficial but not sufficient. In addition, the actual sensor area needs to be as small as possible and needs to be positioned as closely as possible to the surface of the stray field sample in order to maximize the lateral resolution. Here, GFMs with tunable properties fabricated by focused electron beam induced deposition (FEBID) are very attractive. FEBID is a direct-write nanofabrication approach based on the electron-induced dissociation of precursor adsorbates provided by a gas injection system (GIS) inside of a scanning electron microscope (SEM); see [10,11] for recent reviews. In pioneering work by Gabureac and collaborators it was shown that highly miniaturized GFM Hall crosses written by FEBID employing the precursors Co${}_{2}$(CO)${}_{8}$ and hydrocarbons from the residual gas of the SEM can reach stray field sensitivities below 10 μT/$\sqrt{\mathrm{Hz}}$ under ideal conditions, albeit for current levels which are not compatible with sensor areas as small as 50×50 nm${}^{2}$ range [12]. Additionally, in independent work by Cordoba et al. it was shown that GFM prepared by FEBID with the precursor Fe${}_{2}$(CO)${}_{9}$ in the presence of residual water provided by a leak valve can be systematically tuned towards a strongly enhanced EHE [13].

Here we show results following a novel ansatz in which we combine two precursors, HCo${}_{3}$Fe(CO)${}_{12}$ and Me${}_{3}$CpMePt(IV) supplied by two independent gas injectors, to fabricate highly miniaturized granular ferromagnetic Hall crosses with in situ tunable electronic properties. Thereby our focus is on the fabrication process and materials characterization. First results of using the granular ferromagnetic Hall sensors in scanning Hall probe microscopy are presented as a proof-of-principle. We show that post-growth irradiation, an effective method to increase the electrical conductivity of FEBID nano-granular structures fabricated from Me${}_{3}$CpMePt(IV) by several orders of magnitude [14,15], is also applicable to deposits obtained from the Co-Fe and Pt precursor mixture. In addition, we demonstrate that the magnetic field immanent to the objective lens of the SEM in immersion mode can be used to measure the Hall voltage in situ. This opens up an efficient way to find optimized conditions for sensor deposition and post-treatment resulting in the best possible signal-to-noise ratio. First results of scanning Hall probe imaging with these sensors are presented.

## 2. Results and Discussion

### 2.1. Sample Composition and Transport Regime

In order to gain a broad overview of the electronic transport property variations due to changes of composition in granular FEBID samples obtained from mixing the precursors HCo${}_{3}$Fe(CO)${}_{12}$ and Me${}_{3}$CpMePt(IV), the respective gas injection needle positions were varied at fixed precursor temperatures (see also section *Materials and Methods*).

**Figure 1:**
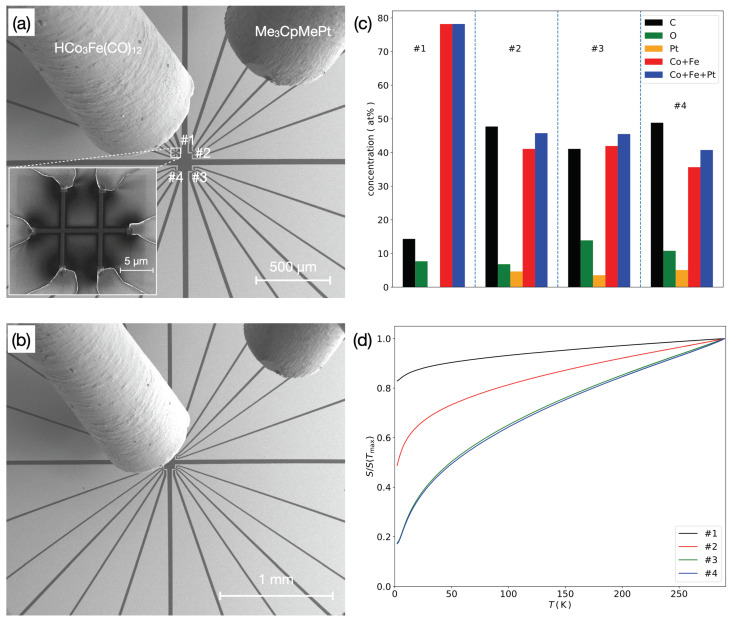
(**a**) SEM image of deposition region showing two gas injectors for the the precursor as indicated. Inset: Enlarged view of deposition area #1 with Cr/Au six-probe electrode structure and granular ferromagnet (GFM) deposit (dark contrast). (**b**) SEM image of injectors with Pt-precursor injector retracted for lower Pt content of GFM deposit. (**c**) GFM composition from EDX analysis of four different samples deposited on one chip at the four positions indicated in (**a**). (**d**) Plot of temperature-dependent conductance normalized to values at 295 K for four GFM samples as indicated.

In Figure 1a a representative geometrical arrangement of the two injectors is shown being positioned above a Si/SiO${}_{2}$ substrate surface subdivided in four quadrants with six-probe Au/Cr electrode structures. For fixed injector positions the resulting four GFM deposits revealed different elemental compositions, as deduced from energy-dispersive X-ray spectroscopy (EDX) indicated in Figure 1c. By changing the injector needles’ distance ratio, as is shown in Figure 1b, another set of four samples was obtained; here with reduced overall Pt content. In the composition example shown in Figure 1c the Co-Fe-precursor injector was positioned very close to the deposition area (distance about 100 μm), whereas the Pt-precursor injector was retracted to a distance of about 1 mm. As a result, the deposit composition of sample #1—closest to the Co-Fe-precursor injector—showed only trace amounts of Pt. The strong injector position sensitivity of the material composition is due to the combination of two effects: (i) the precursor flux density depends sensitively on the distance, see e.g., [16]; (ii) the precursor dynamics when using two precursor species in parallel depends on the details of the chosen preparation parameters, such as dwell time and flux density ratios, see, e.g., [17].

As is apparent from the plot of the temperature-dependent conductance data normalized to the respective values at 295 K, all four GFM samples were in the quasi-metallic regime, i.e., they showed decreasing conductance as the temperature lowered, but the conductance extrapolated to a finite value for T→0; see [18] for a more detailed discussion of the transport regimes of granular metals.

The results on the magneto-transport properties of the GFM Co-Fe/Pt samples shown in the following subsection all refer to the same set of four samples. Within this sample series, the sample numbers #1 to #4 indicated a faster conductance drop as the temperature lowered. For samples #3 and #4 this difference was very subtle but became more apparent in the magneto-transport properties shown next.

### 2.2. Magneto-Transport Properties

#### 2.2.1. Magneto-Resistance and Hall Effect

**Figure 2:**
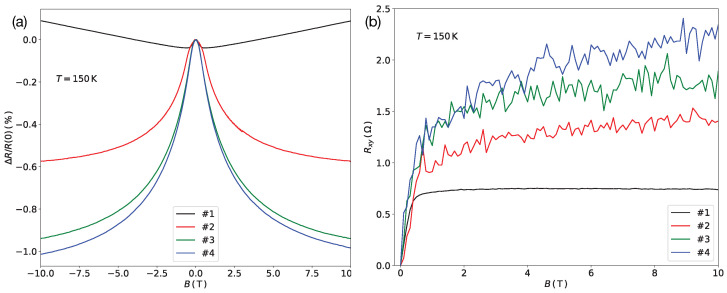
(**a**) Transversal magneto-resistance and (**b**) Hall resistance of samples #1 to #4. Data taken at 150 K under dc current conditions (I=5μA). $B=\mu_{0}H$, with *H* being the applied magnetic field.

In Figure 2 we show selected low-temperature (a) transversal magneto-resistance ΔR/R and (b) Hall resistance $R_{xy}$ data, taken at 150 K under constant current conditions. The magneto-resistance, ΔR/R=(R(B)−R(0))/R(0) with $B=\mu_{0}H$ being the external magnetic flux density applied perpendicularly to the sample surface, showed non-saturating behavior up to 10 T. For the almost Pt-free sample #1 the shape of the magneto-resistance curve was indicative of the anisotropic magneto-resistance effect (AMR) combined with a positive background magneto-resistance, which may be due to bandstructure effects. This same behavior has been found previously in Co-Fe FEBID structures [19]. For samples #2 to #4 spin-dependent inter-grain tunneling was increasingly dominating. The overall magneto-resistance was negative and non-saturating, in particular for samples #3 and #4. Very similar behavior has been found in GFM Fe FEBID structures by Cordoba et al. who made a detailed analysis of the magneto-transport behavior over the temperature range from 10 to 300 K [13]. Here the focus is on optimizing the GFM material properties at room temperature for scanning Hall probe applications.

Turning now to the Hall resistance at 150 K, as shown in Figure 2b, we make three observations: (i) The Hall resistance of the almost Pt-free sample #1 was in qualitatively good agreement with previous results obtained on pure Co-Fe samples [19] and exhibited fully saturated behavior above about 0.5 T, as is expected for a ferromagnet. As compared to the previous results the saturation field reduced by about a factor of two, which finds its natural explanation in the lower Co-Fe metal content and thus reduced saturation magnetization. (ii) For samples #2 to #4 the Hall resistance at large fields exhibited an increasing tendency for non-saturation, indicative of super-paramagnetic behavior, and was enhanced by a factor of up to three (sample #4). This is to be expected for a GFM and shows how the Hall effect can be tuned in this Co-Fe/Pt system by changes in the elemental composition. (iii) The noise level in the Hall resistance data of samples #2 to #4 exemplified the influence of the increasing resistance of the GFM as the overall metal content was reduced. The unavoidable voltage noise

(2) $$V_{th}=\sqrt{4k_{B}TR_{p}\Delta f}$$

caused by the resistance of the voltage probes $R_{p}$ is detrimental to the achievable signal-to-noise ratio (*SNR*) given by

(3) $$SNR=\frac{V_{H}}{V_{th}}=\frac{R_{H}IB}{\sqrt{4k_{B}TR_{p}\Delta f}}\;.$$

In the equations $k_{B}$ is the Boltzman constant, Δf represents the frequency bandwidth, $V_{H}$ is the Hall voltage, $R_{H}=R_{xy}/B$ is the Hall constant and *I* is the current.

The consequences of the noise level with regard to the minimal detectable stray field variation $\delta B_{min}$ will be discussed below. We also note that a reduction in the noisy volume, i.e., the volume of the individual metallic grains, and the reduction in the average density of mobile charge carriers with reducing the metal content will tend to increase the noise power spectral density. We refer to [20] for a more detailed discussion of this behavior in granular Pt FEBID samples.

#### 2.2.2. Scaling Behavior

**Figure 3:**
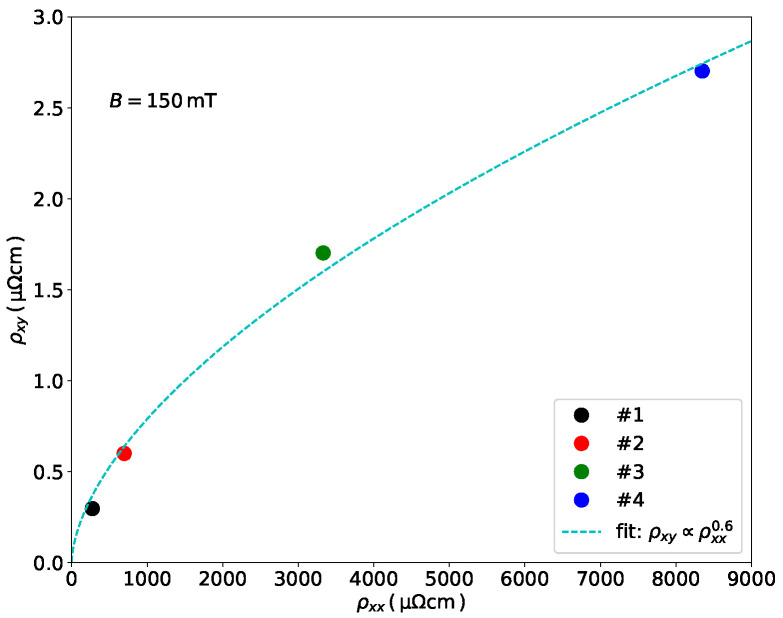
Hall resistivity $\rho_{xy}$ vs. longitudinal resistivity $\rho_{xx}$ as deduced from the data of samples #1 to #4 taken at room temperature in an applied magnetic flux density of 150 mT.

In this subsection we briefly discuss the dependence of the Hall resistivity $\rho_{xy}$ on the longitudinal resistivity $\rho_{xx}$ and analyze whether a scaling behavior of the form $\rho_{xy}\propto \rho_{xx}^{\gamma }$ does occur in our samples. With focus on the use of GFM for magnetic stray field sensing applications we consider, in particular, the scaling behavior at low fields for which saturation of the Hall voltage has not yet occurred. In Figure 3 the Hall resistivity at 150 mT is shown for samples #1 to #4 vs. the respective longitudinal resistivity, as measured at room temperature. A least-squares fit yields $\rho_{xy}\propto \rho_{xx}^{0.6}$, which is indicated as a dashed line in the figure. In a model of additive intrinsic and extrinsic contributions to the Hall effect different types of scaling behaviors can occur [21]. The intrinsic contribution is a consequence of the topological nature of the Bloch states contributing to coherent charge transport. Extrinsic contributions arise due to asymmetric spin-orbit scattering of the spin-polarized electrons on impurities in the ferromagnetic metal. They can lead to linear $\left(\rho_{xy}\propto \rho_{xx}\right)$ or quadratic $\left(\rho_{xy}\propto \rho_{xx}^{2}\right)$ scaling behaviors [8]. It is, however, important to note that the model assumes a ferromagnetic metal spanning the range from weak to strong disorder, i.e., from coherent to diffusive transport. Here charge transport was dominated by inter-granular tunneling for samples #2 to #4 and not by coherent or diffusive transport. In this regime significant deviations from the predicted scaling behavior have been observed in previous research, see, e.g., [22]. In a comprehensive study of the Hall effect in granular Ni/SiO${}_{2}$ in the range from metallic to strongly insulating, Bartov et al. found the Hall resistivity to follow a sub-linear scaling behavior in the (dirty) metal regime with a crossover to a resistivity range in which the Hall resistivity was constant (weakly insulating regime) [23]. Bartov and collaborators also argued that this absence of scaling correlates with a corresponding logarithmic temperature dependence of the conductivity of the Ni/SiO${}_{2}$ samples. In addition, from the theoretical side a breakdown of the scaling behavior $\rho_{xy}\propto \rho_{xx}^{\gamma }$ was predicted for ordered arrays of magnetic nanoparticles in two and three dimensions in the weakly insulating regime [24]. Here, we observed the same type of logarithmic temperature dependence (see Figure 1d) and sub-linear scaling of the Hall resistivity (samples #2 to #4), which may be indicative of the crossover from the bad metal to weakly insulating regime. We note, however, that the additional presence of Pt in the deposits in conjunction with its magnetic polarizability and tendency for alloying with Fe and Co [25,26] does not allow for a simplistic comparison of our results with results obtained from mono-component granular ferromagnets. We therefore kept our focus on the consequences of the observed sub-linear scaling for the performance of the GFM Hall structures for magnetic stray field sensing, and we continue in the next subsection with measures for optimizing the stray field sensitivity by in situ resistance and Hall voltage measurements.

#### 2.2.3. In Situ Measurements and Optimization

**Figure 4:**
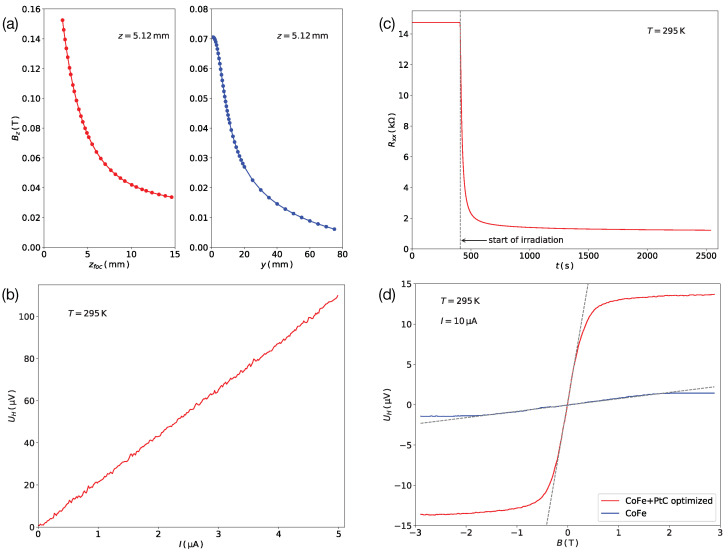
(**a**) Dependence of magnetic induction perpendicular to sample surface at a fixed working distance of 5.12 mm vs. change of focal length of immersion lens (left). Dependence of the magnetic induction for lateral movement at fixed working distance, as indicated, and for fixed focal length (right). (**b**) In situ measured Hall voltage of Co-Fe/Pt GFM Hall cross vs. current. Data taken at room temperature. (**c**) Change of longitudinal resistance $R_{xx}$ vs. time during post-growth electron irradiation of GFM sample at 1.6 nA and 5 keV. (**d**) Comparison of Hall voltage vs. magnetic field measured ex situ for optimized GFM Hall sensor and pure Co-Fe deposit at temperature and current as indicated.

Fabrication of the GFM by FEBID affords in situ measurement of the deposits regarding their transport properties. This has been previously used for FEBID samples in different application fields, see, e.g., [14]. Here, we developed this approach further to include the measurement of magneto-transport properties taking advantage of the magnetic field generated by the objective lens of the SEM in immersion mode. In this mode the magnetic flux density $B_{z}$ perpendicular to the sample surface can be varied by either changing the working distance, i.e., the distance between the objective lens exit aperture and the sample, or the focal length. This is shown in Figure 4a. The field data were calibrated using a commercial Hall sensor mounted to the sample stage of the SEM. In the next step we used the option of periodically modulating the immersion lens current to allow for a lock-in based measurement of the Hall voltage, which works even during the post-growth irradiation process of the GFM Co-Fe/Pt Hall crosses. In Figure 4b we show as an exemplary result the linear dependence of the Hall voltage of a GFM Co-Fe/Pt Hall cross-amplified by a factor of 100 using a low-noise pre-amplifier vs. the measurement current at a fixed immersion lens field of 150 mT.

By tuning the resistance of the Hall sensor using post-growth irradiation, see Figure 4c, the Hall signal can be optimized towards the best possible SNR. In Figure 4d we show the results of ex situ Hall voltage measurements taken on an optimized Hall sensor (red line) in comparison to a pure Co-Fe FEBID sample. From the slope inside the linear response range of about ±200 mT the stray field sensitivity of this particular Hall cross can be deduced and amounted to 3.84 nV/(mT μA). The strong increase in the slope $R_{H}$ of the Hall voltage vs. the applied field demonstrates the associated strong increase in the minimal stray field $\delta B_{min}$ detectable using such an optimized GFM Hall sensor [12]

(4) $$\delta B_{min}=\frac{\sqrt{4k_{B}TR_{p}\Delta f}}{R_{H}\times I_{max}}$$

where $I_{max}$ is the maximum current to which the Hall sensor can be subjected. In the present case, we found that Co-Fe/Pt GFM Hall crosses of 50 nm width and about 30 nm thickness could carry currents of up to $I_{max}=200\;\mu$A at room temperature without heating effects and current-induced irreversible property changes. At this current level the stray field sensitivity amounted to 7.5 μT/$\sqrt{\mathrm{Hz}}$, which is competitive with current state-of-the-art scanning Hall probe sensors based on 2DEGs or semi-metals. Equipped with these insights, we now turn to first results on doing scanning Hall probe microscopy with GFM Co-Fe/Pt Hall cross sensors.

### 2.3. Scanning Hall Probe Microscopy

#### 2.3.1. Probe Geometry

**Figure 5:**
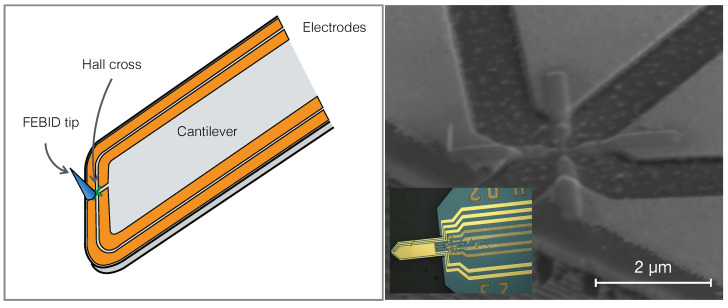
(**Left**): Schematic front edge of self-sensing cantilever with pre-fabricated Cr/Au electrodes between which a GFM Hall sensor cross has been written by FEBID, as well as a non-magnetic tip for complementary atomic force microscopy. (**Right**): SEM image of GFM Hall cross between Cr/Au electrodes. The Hall cross has an effective sensing area of 50×50 nm${}^{2}$. Inset: Optical microscopy image of self-sensing cantilever with cantilever chip.

For scanning Hall probe microscopy measurements a Hall sensor with optimized composition was written onto a self-sensing AFM cantilever with piezoresistive deflection readout. For connection the GFM Hall cross Cr/Au electrodes were patterned in an optical lithography processing step. An optical microscope image is shown in the inset of Figure 5 (right). In order to keep the voltage probes’ lengths short and allow for a small Hall cross-area, the Cr/Au electrodes were initially continuously connected after the lithography step and were then separated by Ga focused ion beam cutting. Into the thus obtained insulating area between the electrodes the GFM Hall sensor was deposited, as shown in Figure 5 (right). A schematic of this cantilever design is depicted in Figure 5 (left). The Hall crossing bars (50×50 nm${}^{2}$) form the area which is sensitive to the orthogonal magnetic stray field generated by a magnetic sample. The FEBID electrodes’ geometry was designed such that it becomes thicker before it makes contact with the Cr/Au electrode edges. This minimizes possible transfer resistances and also somewhat reduces the resistance of the Hall voltage probes for improved signal-to-noise ratios.

In order to measure the anomalous Hall voltage at a constant sensor-sample distance, a tapered granular Pt(C) pillar was deposited by FEBID onto one of the Cr/Au electrodes as close as possible to the front edge of the cantilever. With this pillar used as atomic force microscopy (AFM) tip it was possible to measure the sample’s topography and thus keep the Hall sensor at a fixed distance to the sample surface of about 500 nm. This distance was chosen so as to avoid the risk of crashing the front cantilever edge into the sample surface. With this geometry the lateral resolution of the GFM Hall sensor was limited by the sensor-sample distance to about 500 nm and not the cross-section of the Hall cross. The shift of the tip with regard to the center of the Hall cross introduced a lateral offset of approximately 2 μm between the simultaneously measured topography and magnetic stray field. We comment on possible future improvements of the probe geometry in the discussion section.

#### 2.3.2. Imaging Results

**Figure 6:**
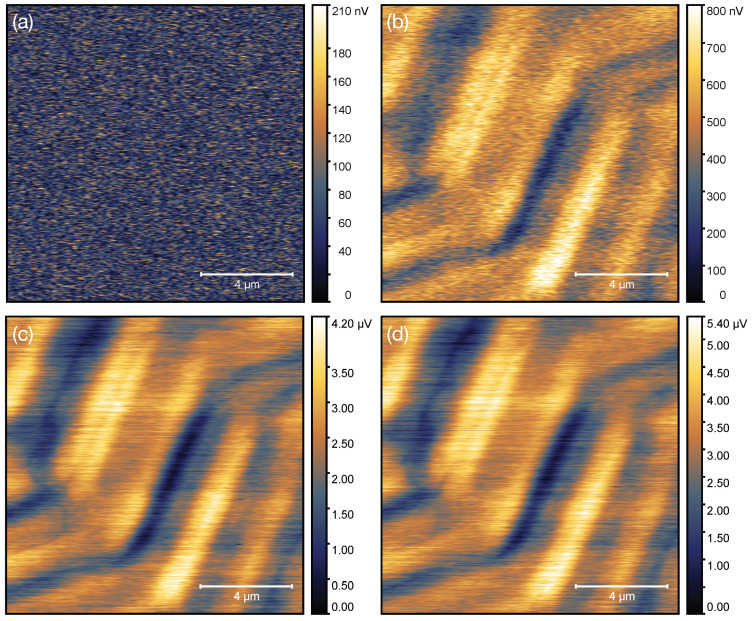
(**a**–**d**) SHPM images recorded with the GFM Hall sensor shown in Figure 5. The ac reference current is increased from 0 μA in (**a**), over 10 μA in (**b**) and 70 μA in (**c**) to 95 μA in (**d**) resulting in an increase in the Hall voltage contrast (see color bar range). Because of non-ideal offset voltage compensation, the smallest Hall voltage measured in each scan was used as reference voltage and set to zero in defining the image contrast scale. The sensor was taken from a batch of sensors written under identical conditions for which we measured representative calibration factors of 2.7 nV/(mT μA) (±10%). See text for details.

**Figure 7:**
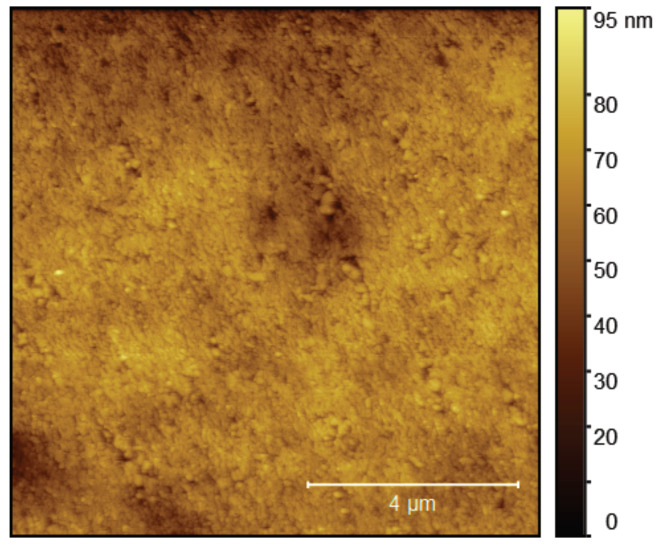
Representative topography image of the magnetic tape taken in vacuum and in dynamic mode for best resolution, whereas AFM images with the SHPM sensor are recorded in static mode. The image was taken at a different position than the SHPM images shown in Figure 6.

In Figure 6 we show results of first proof-of-principle scanning Hall probe measurements with the GFM sensor. They were conducted on a magnetic tape exhibiting bits of different magnetization orientations forming rows in a herringbone arrangement. The topography of the tape was mostly flat with highest features in the order of tens of nanometers, which is apparent from an exemplary topographic image taken in dynamic mode and shown in Figure 7.

The SHPM images shown in Figure 6 were taken with different Hall probe ac currents at 30 kHz modulation frequency at the same sample area. In Figure 6a the Hall signal obtained when scanning without probe current is shown for reference. Except for noise, no contrast was visible, which guarantees that any signal detected for finite probe current was in fact due to the Hall signal generated by the GFM sensor. In Figure 6b–d we demonstrate how the Hall voltage contrast became stronger as the probe current was increased in three steps to 95 μA. At this probe current the maximum Hall voltage contrast was 5.4 μV for this particular sensor over the chosen scan area of 12.9×12.9μ m${}^{2}$. As mentioned before, the lateral magnetic resolution was set by the distance between sample and Hall sensor to about 500 nm in the present case. AC modulation of the Hall probe current at 30 kHz was used in order to reduce 1/f noise contributions, which are known to occur in nanogranular Pt/C [20]. Whether the frequency-dependent voltage noise of granular ferromagnetic CoFe-Pt/C at current density levels up to MA/cm${}^{2}$ follows the same 1/f-type noise characteristics remains for future resolution.

In future work, this could be improved upon by fabricating the GFM sensor element on top of a mesa-like standoff combined with a short tip (50–100 nm) on top of the sensor. This would also reduce the lateral offset between the topographic and magnetic stray field image acquisition, enabling a larger set of samples to be measured and reducing artifacts introduced by steep topographic features.

It will also be important to analyze the long-term stability of the sensor properties regarding their usability in routine scanning Hall probe microscopy. The sensors used in this work have been handled many days and up to weeks under ambient conditions prior to being used in SHPM without significant aging effects regarding the longitudinal and Hall resistivities. This is due to the post-growth electron irradiation for tuning the sensor characteristics, which does also strongly improve the long-term stability. Nevertheless, changes of the resistivity by up to 50% are known to occur on the time scale of one year in irradiated Pt/C deposits [27], and corresponding studies on granular ferromagnetic FEBID structures remain for future investigation.

## 3. Materials and Methods

**Fabrication**. FEBID sample fabrication was done in a dual-beam FIB/SEM microscope (Nova NanoLab 600, FEI Company, Hillsboro, OR, USA) equipped with a Schottky electron emitter. Two standard FEI gas-injection systems (GIS) were used to inject the precursors Me${}_{3}$CpMePt(IV) (Me: methyl, Cp: cyclopentadienyl) and HCo${}_{3}$Fe(CO)${}_{12}$ into the SEM via a capillary with inner and outer diameter of about 0.5 and 0.8 mm each. The temperatures of the precursors were 45 and 65 ${}^{\circ }$C, respectively. The distances from the capillaries to the sample surface were varied between about 100 μm and several mm depending on the targeted sample composition. The base pressure of the microscope chamber was $5\times 10^{-7}$ mbar. The pressure during deposition was about $1.2\times 10^{-6}$ mbar. As substrate material p-doped Si (100) with 200 nm thermally grown SiO${}_{2}$ at room temperature was used for sample property characterization and optimization. The substrates were furnished with Cr/Au electrode structures by standard UV lithography. FEBID writing parameters were 5 kV beam voltage at 1.6 nA beam current with a symmetric pitch of 20 nm in *x*- and *y*-directions and 100 μs dwell time. The patterning strategy was serpentine. To improve the lateral resolution of the Hall sensors on the cantilevers, the beam current and pitch were reduced to 98 pA and 5 nm, respectively. Sample composition was determined by energy-dispersive X-ray spectroscopy (EDX) (EDAX Ametek, Weiterstadt, Germany at 5 kV beam voltage.

**Transport measurements.** Temperature-dependent conductance, magneto-resistance and Hall effect measurements were done in a ${}^{4}$He cryostat (Oxford Instruments NanoScience, Tubney Woods, Abingdon, UK), equipped with a 14 T superconducting solenoid, with variable temperature insert (VTI) operating in the range from 1.6 to 300 K. The conductivity measurements were done in constant voltage mode employing a Keithley 2635B source meter (Keithley/Tektronix, Beaverton, OR, USA) and Agilent 34420A nanovolt meter (Agilent Technologies, Santa Clara, CA, USA). The magneto-resistance and Hall effect measurements at fixed temperature and under magnetic field variation were done in constant current mode. In situ conductance and Hall measurements, i.e., measurements inside of the microscope, were done by a lock-in technique. In particular, for the Hall voltage measurements the lock-in amplifier was synchronized with the periodically modulated magnetic field of the immersion lens of the electron microscope.

**Scanning probe microscopy.** The scanning probe microscopy images were taken with the AFSEM^®^(GETec Microscopy GmbH, Vienna, Austria), an AFM system that can be integrated into scanning electron microscopes [28,29]. A scanning probe microscopy controller (Anfatec Instruments AG, Oelsnitz/Vogtl., Germany) that contains a multi-channel lock-in amplifier was used for the AFM and SHPM measurements. As a platform for SHPM probes, self-sensing cantilevers with integrated piezoresistive elements from AMG Technology Ltd. were used. The sample imaged in the SHPM and AFM measurements was a magnetic digital backup tape from Nanosurf AG. The topography was recorded in vacuum with a pressure of approximately 10${}^{-5}$ mbar and in dynamic mode. The SHPM measurements were conducted in air at normal pressure. The images were taken in static mode, simultaneously acquiring topography and SHPM images. The lock-in reference voltage was set to 30 kHz, which was converted with the AFM electronics into an ac current, so that 1 mV corresponded to 1 μA probe current.

## 4. Conclusions and Outlook

In this work we have demonstrated how in situ tunable, highly miniaturized Hall sensors based on a granular ferromagnet can be fabricated by FEBID employing two precursors providing Co-Fe and Pt in parallel. Hall sensor structures optimized for large extraordinary Hall effect combined with moderately high longitudinal resistivity values were integrated into self-sensing cantilevers for scanning Hall probe microscopy in first proof-of-principle experiments. In these experiments the lateral resolution was limited by the sample sensor distance (≈500 nm) imposed by the probe geometry and not by the lateral size of the Hall sensor itself (50×50 nm${}^{2}$).

In future research, several avenues are worthwhile to pursue. First, with regard to further optimizing the achievable stray field sensitivity, insight into the microstructure of the Co-Fe/Pt granular FEBID structures would be desirable. Considering the expected scale of the nanograins of only a few nanometers this is no simple task. Second, with a view to employing the Co-Fe/Pt structures for scanning Hall probe microscopy on a routine basis, possible issues with long-term stability of the Hall and longitudinal resistivity have to be investigated. Finally, optimizations of the sensor probe geometry would imply the use of mesa-like standoffs for deposition of the Hall sensors, thus allowing to bring the sensor area to a distance of less than 100 nm to the surface of the magnetic sample. The use of circular-shaped Hall sensor areas would help to mitigate possible in-plane shape anisotropy effects associated with the square or rectangular Hall cross-geometry. Replacement of the voltage probe leads, currently made from Co-Fe/Pt, by Cr/Au would help to reduce the resistance noise, albeit at the cost of increased complexity in the overall fabrication process. It is also feasible to reduce the area of the sensing element to below 50 nm, e.g., aiming for 20 nm, which seems feasible with FEBID. At this degree of miniaturization a closer look into possible Hall signal contributions from the co-deposit will be necessary, as this can compromise the ultimately achievable lateral resolution.

## Data Availability

The data presented in this study are available on request from the corresponding author. The data are not publicly available as the data format is not self-explanatory.

## Abbreviations

The following abbreviations are used in this manuscript:

MFM	Magnetic force microscopy
SSQM	Scanning SQUID microscopy
SHPM	Scanning Hall probe microscopy
GFM	Granular ferromagnet
OHE	Ordinary Hall effect
EHE	Extraordinary Hall effect
FEBID	Focused electron beam induced deposition
GIS	Gas injection system
SEM	Scanning electron microcope
EDX	Energy-dispersive X-ray spectroscopy
AFM	Atomic force microscopy
